# Evaluation of a commercial multiplex pathogen panel for the diagnosis of pediatric *Kingella kingae* joint infections

**DOI:** 10.1128/jcm.01039-25

**Published:** 2025-09-03

**Authors:** Philippe Bidet, Stéphane Bonacorsi, Matthis Lingua, Anne-Laure Simon, Marie Parizot, Marion Caseris, André Birgy

**Affiliations:** 1 Service de Microbiologie, Hôpital Robert-Debré, Assistance Publique des Hôpitaux de Paris (AP-HP), Paris, France; 2 IAME, UMR 1137, INSERM 27102, Paris, France; 3 Université Paris Cité 555089, Paris, France; 4 Service d’orthopédie pédiatrique, Hôpital Robert-Debré, Assistance Publique des Hôpitaux de Paris (AP-HP) 26929 , Paris, France; 5 Service de Pédiatrie Générale, Hôpital Robert-Debré, Assistance Publique des Hôpitaux de Paris (AP-HP), Paris, France; Cleveland Clinic, Cleveland, Ohio, USA

**Keywords:** multiplex PCR, children, arthritis, PCR, *Kingella kingae*

## LETTER

We read with interest the manuscript by Copley et al. concerning the performance of BIOFIRE JI Panel (bioMérieux) for the diagnosis of pediatric joint infections (JIs) (1). This study was performed on 63 patients, of whom 24 were 0.5–4 years old (37.5%). Among those young children, three were positive for *Kingella kingae* both by the multiplex panel and by 16S-rDNA PCR.

Diagnosis of *K. kingae* JI is clinically relevant, since they have a better prognosis than those caused by *Staphylococcus aureus*, may benefit from shorter antimicrobial therapy with the potential use of amoxicillin alone in countries where beta-lactamase producing strains are rare and avoids further biological tests exploring non-infectious etiologies (2–4).

*K. kingae* is a fastidious bacterium, and culture of joint samples is rarely positive. Most diagnoses are done by PCR, especially by specific PCR, which is less time-consuming, easier to implement, and more sensitive than 16S-rDNA PCR (3–5).

Thus, 16S-rDNA PCR, although more sensitive than culture, does not perform as well as specific *K. kingae* PCR, and the latter should preferentially be used as a comparator for evaluating new diagnostic tools for JI in young children.

When *K. kingae*-specific PCR is implemented for the diagnosis of pediatric JI, the rate of positive diagnosis is about one-third (2). Considering only the 24 children aged 0.5–4 years in this study, the number of cases of *K. kingae* arthritis diagnosed appears less than expected (1).

To further evaluate the sensitivity of the BIOFIRE JI Panel for the specific diagnosis of *K. kingae* arthritis, we have thus undertaken a comparative study with a specific *K. kingae* PCR (KING-UX, Progenie molecular, Valencia, Spain) on 33 joint fluids of 32 children aged 7 months to 5 years suspected of bacterial arthritis between September 2022 and April 2024.

Among the samples included, 24 out of 33 (68%) were positive using the specific *K. kingae* PCR, of which only 19 were also positive for *K. kingae* target using the BIOFIRE JI Panel (Table 1). The five samples not detected by the panel had a cycle threshold (CT) >33 in the specific PCR. To further study the sensitivity of the BIOFIRE JI Panel, we performed serial dilutions of both a positive sample and of *K. kingae* ATCC23331 and confirmed that the dilutions detected by the King-UX PCR with a CT >33 were not amplified by the panel. To evaluate if the type of container used for joint fluids (either EDTA or Sodium-Heparinate) could hamper the performance of the panel, we incubated serial dilutions of the *K. kingae* strain with both anticoagulants and obtained the same results with similar CT values (Table 1).

**TABLE 1:** Results of *K. kingae* culture and PCR of joint fluid pediatric samples by BIOFIRE JI panel and simplex PCR (KING-UX)^*a*^

Sample	CT BIOFIRE JI Panel	CT KING-UX PCR	Culture	Age	Sample date
22670072	NEG	36	NEG	1 y	24/09/2022
22710153	11.8	30	NEG	1 y	28/09/2022
22840170	NEG	NEG	NEG	4 y	11/10/2022
22980111	11	27	NEG	1 y	25/10/2022
23060133	14	31	NEG	1 y	02/11/2022
23060141	NEG	NEG	NEG	2 y	02/11/2022
23110092	NEG	NEG	NEG	3 y	07/11/2022
23340184	10	27	NEG	2 y	30/11/2022
23340135	13	26	NEG	9 m	30/11/2022
23490181	13	26	POS	1 y	15/12/2022
23530172	19.4	33	NEG	7 m	19/12/2022
23540190	19	33.7	NEG	1 y	20/12/2023
23560109	NEG	35.7	NEG	8 m	22/12/2023
23560110	17	32.6	NEG	8 m	22/12/2023
23600099	NEG	NEG	NEG	2 y	26/12/2023
23600104	NEG	NEG	NEG	2 y	26/12/2023
23600151	NEG	NEG	NEG	1 y	26/12/2023
30010046	NEG	36.2	NEG	1 y	01/01/2023
30170130	NEG	35.6	NEG	11 m	17/01/2023
30170135	NEG	NEG	NEG	4 y	17/01/2023
30300064	14	28	NEG	1 y	30/01/2023
30510106	9	25	NEG	1 y	20/02/2023
30510108	16	30	NEG	2 y	20/02/2023
30460004	12	27	POS	11 m	15/02/2023
231334564601	11	30	NEG	11 m	29/11/2023
231331294001	15	30	NEG	1 y	02/03/2022
240020729901	NEG	33.5	NEG	1 y	20/01/2024
240058004001	NEG	NEG	NEG	4 y	23/02/2024
240048154501	NEG	NEG	NEG	5 y	14/02/2024
240082566801	17.6	31.4	POS	2 y	18/03/2024
240096238201	14.1	29.7	NEG	1 y	31/03/2024
240031777901	16.3	31.4	NEG	2 y	30/01/2024
240125188401	13.5	33.5	NEG	1 y	29/04/2024
Diluted sample					
23530172	16	30			
23530172 1/10	NEG	36.5			
23530172 1/100	NEG	37.6			
Strain dilution					
K kingae ATCC23331 (4 CFU/mL)	NEG	36.5			
K kingae ATCC23331 (2 CFU/mL)	NEG	37.6			
Strain dilution (1 mL) in either EDTA or Heparinate tube					
1000 CFU/mL in EDTA	12.4				
1000 CFU/mL in Heparinate	12.8				
100 CFU/mL in EDTA	14.6				
100 CFU/mL in Heparinate	15.3				
10 CFU/mL in EDTA	18.4				
10 CFU/mL in Heparinate	19				

^*a*^ NEG: negative, POS: positive. For positive PCR results, the CT value alone is indicated. The CT of the BIOFIRE JI panel is that of the second amplification (nested PCR). Ages are in year (y) or months (m).

Thus, orthopedists and pediatricians should be alerted that BIOFIRE JI Panel, although very easy to implement for the detection of *K. kingae* with excellent specificity, has a sensitivity of about 79% and that samples of children aged 5 months to 5 years negative for this target should be further tested by a specific monoplex PCR before ruling out this diagnosis.

## References

[1] Copley L, Lee G, Villani M, Tareen N, Filkins LM. 2025. Performance evaluation of a commercial multiplex pathogen panel for the diagnosis of pediatric joint infections. J Clin Microbiol 63:e0027825. doi:10.1128/jcm.00278-25 40454835 PMC12239719

[2] Ilharreborde B, Bidet P, Lorrot M, Even J, Mariani-Kurkdjian P, Liguori S, Vitoux C, Lefevre Y, Doit C, Fitoussi F, Penneçot G, Bingen E, Mazda K, Bonacorsi S. 2009. New real-time PCR-based method for Kingella kingae DNA detection: application to samples collected from 89 children with acute arthritis. J Clin Microbiol 47:1837–1841. doi:10.1128/JCM.00144-09 19369442 PMC2691089

[3] Yagupsky P. 2017. Diagnosing Kingella kingae infections in infants and young children. Expert Rev Anti Infect Ther 15:925–934. doi:10.1080/14787210.2017.1381557 28918656

[4] Chometon S, Benito Y, Chaker M, Boisset S, Ploton C, Bérard J, Vandenesch F, Freydiere AM. 2007. Specific real-time polymerase chain reaction places Kingella kingae as the most common cause of osteoarticular infections in young children. Pediatr Infect Dis J 26:377–381. doi:10.1097/01.inf.0000259954.88139.f4 17468645

[5] Morel A-S, Dubourg G, Prudent E, Edouard S, Gouriet F, Casalta J-P, Fenollar F, Fournier PE, Drancourt M, Raoult D. 2015. Complementarity between targeted real-time specific PCR and conventional broad-range 16S rDNA PCR in the syndrome-driven diagnosis of infectious diseases. Eur J Clin Microbiol Infect Dis 34:561–570. doi:10.1007/s10096-014-2263-z 25348607

